# The prognostic value of [^18^F]FDG PET/CT based response monitoring in metastatic melanoma patients undergoing immunotherapy: comparison of different metabolic criteria

**DOI:** 10.1007/s00259-023-06243-y

**Published:** 2023-04-26

**Authors:** Christos Sachpekidis, Vivienn Weru, Annette Kopp-Schneider, Jessica C. Hassel, Antonia Dimitrakopoulou-Strauss

**Affiliations:** 1 Clinical Cooperation Unit Nuclear Medicine, German Cancer Research Center (DKFZ), Im Neuenheimer Feld 280, 69210 Heidelberg, Heidelberg, Germany; 2 Department of Biostatistics, German Cancer Research Center (DKFZ), Heidelberg, Germany; 3 Department of Dermatology and National Center for Tumor Diseases (NCT), University Hospital Heidelberg, Heidelberg, Germany

**Keywords:** Metastatic melanoma, Immune checkpoint inhibitors (ICIs), Immunotherapy, Treatment response evaluation, [^18^F]FDG PET/CT, Metabolic response criteria

## Abstract

**Purpose:**

To investigate the prognostic value of [^18^F]FDG PET/CT as part of response monitoring in metastatic melanoma patients treated with immune checkpoint inhibitors (ICIs).

**Methods:**

Sixty-seven patients underwent [^18^F]FDG PET/CT before start of treatment (baseline PET/CT), after two cycles (interim PET/CT) and after four cycles of ICIs administration (late PET/CT). Metabolic response evaluation was based on the conventional EORTC and PERCIST criteria, as well as the newly introduced, immunotherapy-modified PERCIMT, imPERCIST5 and iPERCIST criteria. Metabolic response to immunotherapy was classified according to four response groups (complete metabolic response [CMR], partial metabolic response [PMR], stable metabolic disease [SMD], progressive metabolic disease [PMD]), and further dichotomized by response rate (responders = [CMR] + [PMR] vs. non-responders = [PMD] + [SMD]), and disease control rate (disease control = [CMR] + [PMR] + [SMD] vs. [PMD]). The spleen-to-liver SUV ratios (SLR_mean_, SLR_max_) and bone marrow-to-liver SUV ratios (BLR_mean_, BLR_max_) were also calculated. The results of PET/CT were correlated with patients’ overall survival (OS).

**Results:**

Median patient follow up [95% CI] was 61.5 months [45.3 – 66.7 months]. On interim PET/CT, the application of the novel PERCIMT demonstrated significantly longer survival for metabolic responders, while the rest criteria revealed no significant survival differences between the different response groups. Respectively on late PET/CT, both a trend for longer OS and significantly longer OS were observed in patients responding to ICIs with metabolic response and disease control after application of various criteria, both conventional and immunotherapy-modified. Moreover, patients with lower SLR_mean_ values demonstrated significantly longer OS.

**Conclusion:**

In patients with metastatic melanoma PET/CT-based response assessment after four ICIs cycles is significantly associated with OS after application of different metabolic criteria. The prognostic performance of the modality is also high after the first two ICIs cycles, especially with employment of novel criteria. In addition, investigation of spleen glucose metabolism may provide further prognostic information.

**Supplementary Information:**

The online version contains supplementary material available at 10.1007/s00259-023-06243-y.

## Introduction

The recent advent and success of novel immunotherapies has undoubtedly constituted the paramount achievement in cancer treatment, leading to a paradigm shift in the field of oncology [1]. In particular in advanced and metastatic melanoma, the clinical application of immune checkpoint inhibitors (ICIs) in the last decade has been a major breakthrough in the management of the disease, leading to unprecedented response and survival rates [2-4]. The usage of ICIs is considered nowadays the standard practice for the treatment of metastatic melanoma [5].

ICIs act markedly different than conventional cytotoxic approaches, such as chemotherapy, notably through the stimulation of the immune response, subsequent generation of inflammation, and reduction of tolerance of the immune system to tumor cells, rather than direct tumor lysis. In specific, these agents bind to the immune checkpoints, mainly cytotoxic T-lymphocyte-associated protein 4 (CTLA-4), programmed cell death protein 1 (PD-1) or its ligand, programmed cell death ligand 1 (PD-L1), leading to unleashing of T lymphocyte-mediated immune responses against tumors [4, 6]. This unique mechanism of action of cancer immunotherapies may lead to novel, atypical response patterns, which have been observed only seldom following chemotherapy or targeted therapies. Such patterns include the phenomena of pseudoprogression, hyperprogression, dissociated and delayed responses, and may pose challenges in the interpretation of treatment response by conventional imaging approaches [7-9]. Moreover, in the context of the activation of the host immune system triggered by ICIs, collateral, inflammatory side effects, addressed as immune-related adverse events (irAEs), often occur, whose early recognition represents another challenge for patient management and prognosis, due to the suggested intimate link between their emergence and the anti-tumor effect elicited by ICIs [10-12].

In this context, the reliable assessment of response to these expensive and potentially toxic agents early in the course of treatment would offer significant therapeutic and prognostic implications in the entire spectrum of patient management. [^18^F]FDG PET/CT may serve as a reliable method for patient stratification and response monitoring to ICIs, with several recent studies demonstrating its potential in the characterization of the response patterns observed with immunotherapy [8, 13, 14]. These attempts have led to the development of new, modified, metabolic response criteria for capturing the atypical patterns of tumor response [15-18]. The main novelty introduced by these criteria concerns the definition of progressive disease, for the identification of which a simple emergence of a new lesion is not sufficient. In the same framework, different PET biomarkers, derived from both tumoral and non-tumoral tissue, have been proposed for prediction of response to ICIs [19-23]. Nevertheless, both the most appropriate evaluation approach and the optimal time-points for PET/CT scanning during the course of immunotherapy remain to be defined, as underlined in the recently published guidelines on the use of [^18^F]FDG PET/CT imaging during cancer immunomodulatory treatments [24].

In view of the previous considerations, aim of the present study was to investigate the prognostic value of [^18^F]FDG PET/CT as part of response monitoring in metastatic melanoma patients treated with ICIs. In particular, we studied the relationship of tumor response on [^18^F]FDG PET/CT, applied at different time points during immunotherapy, and evaluated by different metabolic criteria, and survival of these patients. The relation between potential signs of immune activation on [^18^F]FDG PET/CT and long-term patient outcome was also assessed.

## Materials and Methods

### Patients

Sixty-seven patients (44 males, 23 females; mean age 56.9 years) with unresectable, stage IV melanoma undergoing immunotherapy with ICIs were enrolled in this retrospective analysis of a prospectively conducted study. Some of these patients have been analyzed in other publications but with different approaches than in the here presented study [16]. Patients received CTLA-4 inhibitors (ipilimumab), PD-1 inhibitors (pembrolizumab, nivolumab) or a combination of CTLA-4 and PD-1 inhibitors (nivolumab/ipilimumab). Ipilimumab was administered intravenously at a dose of 3 mg/kg every 3 weeks for a total of 4 doses. Pembrolizumab was administered intravenously at a dose of 2 mg/kg every 3 weeks. Nivolumab was administered intravenously at a dose of 3 mg/kg every 2 weeks. The combination ICI therapy was administered as an induction of 4 cycles of nivolumab (1 mg/kg) and ipilimumab (3 mg/kg) every 3 weeks, followed by single-agent nivolumab administration (3 mg/kg) every 2 weeks. The included patients had not received chemotherapy for at least one month prior to the initial PET/CT studies. None of the patients had a history of diabetes. Patients gave written informed consent to participate in the study and to have their medical records released. The study was approved by the Ethical Committee of the University of Heidelberg (S-107/2012) and the Federal Agency for Radiation Protection (Bundesamt für Strahlenschutz, Z 5 – 22,463/2 – 2012–016).

### [^18^F]FDG PET/CT data acquisition

[^18^F]FDG PET/CT was performed before the start of treatment (baseline PET/CT), after two cycles (interim PET/CT; 4–6 weeks after initiation of treatment) and after four cycles of ICIs’ administration (late PET/CT; 8–12 weeks after initiation of treatment) in all patients.

Patients underwent a whole body PET/CT after intravenous administration of maximum 250 MBq [^18^F]FDG 60 min post-injection (p.i.). Imaging was performed from the head to the feet with an image duration of 2 min per bed position. A dedicated PET/CT system (Biograph mCT, S128, Siemens Co., Erlangen, Germany) with an axial field of view of 21.6 cm with TruePoint and TrueV, operated in a three-dimensional mode was used. A low-dose attenuation CT (120 kV, 30 effective mAs) was used for attenuation correction of the PET data and for image fusion. All PET images were attenuation-corrected and an image matrix of 400 × 400 pixels was used for iterative image reconstruction. Iterative image reconstruction was based on the ordered subset expectation maximization (OSEM) algorithm with point spread function modelling (2 iterations and 21 subsets) as well as time of flight (TOF).

### [^18^F]FDG PET/CT data analysis

PET/CT images were analyzed and interpreted in consensus by two experienced nuclear medicine physicians well versed in melanoma diagnosis (CS, ADS). In visual analysis, an [^18^F]FDG–avid lesion was defined as focal, abnormally increased tracer uptake versus background activity, with or without a corresponding anatomic lesion on the CT scan, and consistent with tumor/metastasis. The detailed characteristics of target lesion definition according to the different applied metabolic criteria are described in the following section and in Table 1.

**Table 1:** Summary of the herein applied metabolic criteria for assessment of response to immunotherapy

	EORTC, 1999	PERCIST, 2009	PERCIMT, 2018	imPERCIST5, 2019	iPERCIST, 2019
Target lesion definition	Tumor lesion with the highest SUV uptake	Minimum tumor SUL 1.5 * mean SUL liver + 2SDs, ≤ 5 target lesions/patient	Circumscribed sites of non-physiological [^18^F]FDG uptake greater than surrounding background	Minimum tumor SUL 1.5 * mean SUL liver + 2SDs, ≤ 5 target lesions/patient	Minimum tumor SUL 1.5 * mean SUL liver + 2SDs, ≤ 5 target lesions/patient
New lesions	PMD	PMD	Cut-off of four new lesions for PMD of any size	Need to be included in the sum of SULpeak if they show higher uptake than existing target lesions; PMD if > 30% increase in sum of SULpeak	Unconfirmed PMD (uPMD)
CMR	Complete resolution of [^18^F]FDG uptake within the tumor volume so that it is indistinguishable from surrounding normal tissue	Disappearance of all metabolically active lesions	Complete resolution of all pre-existing [^18^F]FDG avid lesions. No new, [^18^F]FDG avid lesions	Disappearance of all metabolically active lesions	Disappearance of all metabolically active lesions
PMR	A reduction of a minimum of 15–25% in tumor SUV after one cycle of chemotherapy, and > 25% after more than one treatment cycle	Reduction in sum of SULpeak in target lesions > 30% and absolute drop in SUL > 0.8 SUL units	Complete resolution of some pre-existing [^18^F]FDG avid lesions. No new, [^18^F]FDG avid lesions	Reduction in sum of SULpeak in target lesions ≥ 30% and absolute drop in SUL by ≥ 0.8 SUL units	Reduction in sum of SULpeak in target lesions > 30% and absolute drop in SUL > 0.8 SUL units
SMD	An increase in SUV < 25% or a decrease < 15% and no visible increase in extent of [^18^F]FDG tumor uptake (> 20% in the longest dimension)	Neither PMD, PMR, nor CMR	Neither PMD, PMR, nor CMR	Neither PMD, PMR, nor CMR	Neither PMD, PMR, nor CMR
PMD	An increase in SUV > 25% within the tumor region defined on the baseline scan, visible increase in the extent [^18^F]FDG tumor uptake (> 20% in the longest dimension) or the appearance of new [^18^F]FDG uptake in metastatic lesions	Increase in sum of SULpeak of > 30% or the appearance of a new lesion	≥ 4 new lesions of less than 1 cm in functional diameter or ≥ 3 new lesions between 1.0 – 1.5 cm in functional diameter or ≥ 2 new lesions of more than 1.5 cm in functional diameter	> 30% increase in SULpeak, with > 0.8 SUL unit increase in tumor SULpeak	≥ 30% increase in SULpeak or new metabolically active lesions: unconfirmed PMD (uPMD)
Confirmation of PMD	n.a	n.a	n.a	n.a	PET at 4–8 weeks: confirmed PMD (cPMD)

*CMR* complete metabolic response, *PMR *partial metabolic response, *SMD *stable metabolic disease, *PMD *progressive metabolic disease, *SUV *standardized uptake value, *SUL *SUV normalized by the lean body mass, *EORTC *European Organization for Research and Treatment of Cancer, *PERCIST *Positron Emission Tomography Response Criteria in Solid Tumors, *PERCIMT *PET Response Evaluation Criteria for IMmunoTherapy, *imPERCIST5 *immunotherapy-modified PERCIST, *iPERCIST *immune PERCIST, *n.a.* not applicable

Moreover, patterns of [^18^F]FDG uptake on interim and late PET/CT suggestive of manifestations of irAEs to immunotherapy were documented. Given the lack of a standard definition for immunotherapy-induced organ inflammation, PET-irAEs were defined visually as sites of newly emerging or markedly increased compared to baseline imaging, non-malignant [^18^F]FDG accumulation in organs known to exhibit immune-related signs on PET/CT [21, 24-26]. In specific, a diffusely enhanced tracer uptake in the gastrointestinal tract, the thyroid gland, the lungs and the bone marrow, or, respectively, a relatively symmetrical, increased uptake in lymph nodes (e.g. mediastinal/hilar) and in joints following ICIs were considered suggestive of PET-irAEs in these organs.

Semi-quantitative evaluation was based on volumes of interest (VOIs) drawn over melanoma lesions and on subsequent calculation of mean standardized uptake value (SUV_mean_), maximum SUV (SUV_max_) and peak SUV corrected for lean body mass (SUL_peak_). Moreover, the SUV_mean_ and SUV_max_ of the physiologic liver and spleen parenchyma as well the bone marrow were measured after placing a VOI the right liver lobe, centrally in the spleen, and the lower thoracic vertebral bodies, respectively. Based on these measurements, the spleen-to-liver SUV ratios (SLR_mean_, SLR_max_) as well the bone marrow-to-liver SUV ratios (BLR_mean_, BLR_max_) were calculated in all scans. VOIs were drawn using the pseudo-snake algorithm of the Pmod software (http://www.pmod.com/files/download/v31/doc/pbas/4729.htm).

### Target lesion(s) definition

In general, there are two major approaches for definition of target tumor lesions. The first one is based on visual reading of the PET/CT scans, defines tumor lesions as circumscribed sites of increased [^18^F]FDG uptake greater than the surrounding background activity, and does not require a minimum background. This approach is applied in the European Organization for Research and Treatment of Cancer (EORTC) 1999 criteria [27] and the novel PET Response Evaluation Criteria for IMmunoTherapy (PERCIMT) [16]. The second approach is applied in the Positron Emission Tomography Response Criteria in Solid Tumors (PERCIST) [28], the novel Immunotherapy-modified PERCIST (imPERCIST5) [17] as well as the immune PERCIST (iPERCIST) [18], and involves a detailed definition for target lesions, in which SUL_peak_ should be at least 1.5 times greater than the SUL_mean_ + 2SDs of the normal right liver lobe (Table 1).

Due to the different approaches for definition of target lesions applied by the various metabolic response criteria, in the present analysis the target lesions were identified by the following methods:

- Visually/subjectively with the use of the Aycan workstation for EORTC and PERCIMT.
- Automatically with the employment of the Pmod Version 4.1 software for PERCIST, imPERCIST5 and iPERCIST.

### Response evaluation

Patients with at least one [^18^F]FDG–avid lesion consistent with metastasis in visual analysis of the PET/CT scans were assessed according to metabolic response criteria. Specifically, the evaluation of patients’ response to immunotherapy by means of [^18^F]FDG PET/CT was performed after application of the following criteria:

- European Organization for Research and Treatment of Cancer (EORTC) 1999 criteria [27]
- Positron Emission Tomography Response Criteria in Solid Tumors (PERCIST) [28]
- PET Response Evaluation Criteria for IMmunoTherapy (PERCIMT) [16]
- Immunotherapy-modified PERCIST (imPERCIST5) [17]
- Immune PERCIST (iPERCIST) [18] (Table 1).

Based on these criteria, patients’ metabolic response to immunotherapy was defined by:

- The four categories of metabolic response (complete metabolic response [CMR], partial metabolic response [PMR], stable metabolic disease [SMD], or progressive metabolic disease [PMD]).
- Response rate “RR” (responders = [CMR] + [PMR] vs. non-responders = [PMD] + [SMD]).
- Disease control rate “DCR” (disease control = [CMR] + [PMR] + [SMD] vs. no-disease control = [PMD]).

### Statistical analysis

To investigate the potential prognostic role of the different metabolic response evaluation criteria, the emergence of immune-related adverse events on PET/CT as well as quantitative PET parameters derived from physiologic non-tumoral tissues in patient’s overall survival (OS), Kaplan–Meier plots and log-rank test were used. OS was defined for each PET/CT as the time since the respective PET/CT to death or last follow-up. Median follow-up was calculated using the reverse Kaplan–Meier. Statistical analysis was performed using R (version 4.0.3, packages: survival, survminer, prodlim). As the study was exploratory, no correction for multiple testing was performed. The results were considered significant for p-values less than 0.05 (p < 0.05).

## Results

### Patient cohort

All included patients underwent immunotherapy with at least four cycles of ICIs as described above, without disruption of the treatment due to irAEs or other causes during this period. In specific, they received CTLA-4 inhibitors (ipilimumab, *n* = 49 patients), PD-1 inhibitors (pembrolizumab, *n* = 5 patients; nivolumab, *n* = 2 patients) or a combination of CTLA-4 and PD-1 inhibitors (nivolumab/ipilimumab, *n* = 11 patients). Baseline mean LDH was 276.0 U/l (median = 234.0 U/l). Median follow up [95% CI] of the patient cohort from start of treatment was 61.5 months [45.3 – 66.7 months]. At the time of data cutoff for the analysis, 38 patients (56.7%) had died. Median OS for all patients was 37.8 months [21.1 – NA] from start of treatment.

### Analysis of baseline PET/CT findings

At baseline, 61/67 patients of the whole cohort (91%) had at least one [^18^F]FDG–avid lesion consistent with metastasis based on visual analysis of the PET/CT scans. These patients were eligible for further assessment with the metabolic response criteria for immunotherapy. Respectively, the use of the automated approach for identification of target tumor lesions on the basis of the PERCIST approach, led to the detection of at least one lesion in 45/67 patients (67%).

**Fig. 1:**
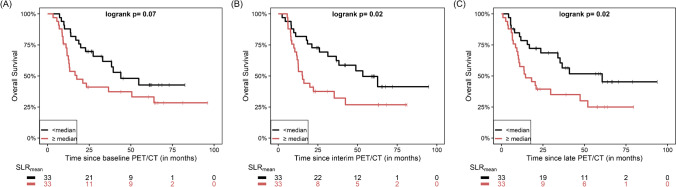
Kaplan–Meier estimates of OS according the SLR_mean_ derived from baseline (**A**), interim (**B**) and late PET/CT (**C**). The numbers of patients at risk in each group and for the respective time-points are shown below the plots

With regard to the quantitative parameters derived from physiologic non-tumoral tissues, SLR values were calculated in 66/67 patients (98.5%), since one patient had undergone splenectomy, while BLR values were calculated in all 67 patients (Table 2). The respective Kaplan–Meier curves demonstrated a trend for longer OS in patients with lower SLR_mean_ values, although the log rank test revealed no statistical significance (p = 0.07) (Fig. 1A). None of the rest quantitative baseline parameters had a significant effect on patient OS (Table 3).

**Table 2:** Descriptive statistics of the quantitative parameters derived from physiologic non-tumoral tissues

Parameter	Baseline PET/CT		Interim PET/CT		Late PET/CT	
	*Mean (SD)*	*Median*	*Mean (SD)*	*Median*	*Mean (SD)*	*Median*
SLR_mean_	0.89 (0.13)	0.87	0.92 (0.17)	0.92	0.94 (0.17)	0.92
SLR_max_	0.86 (0.15)	0.85	0.88 (0.19)	0.83	0.89 (0.18)	0.86
BLR_mean_	1.02 (0.24)	0.98	1.05 (0.28)	1.02	1.13 (0.36)	1.04
BLR_max_	0.99 (0.26)	0.97	1.03 (0.33)	0.98	1.12 (0.36)	1.04

*SLR*_*mean*_ spleen to liver SUV_mean_ ratio, *SLR*_*max*_ spleen to liver SUV_max_ ratio, *BLR*_*mean*_ bone marrow to liver SUV_mean_ ratio, *BLR*_*max*_ bone marrow to liver SUV_max_ ratio

**Table 3:** Results of survival analysis based on quantitative data from physiologic non-tumoral tissues

Parameter		Baseline PET/CT		Interim PET/CT		Late PET/CT	
		Median OS [95% CI]	*p value*	Median OS [95% CI]	*p value*	Median OS [95% CI]	*p value*
SLR_mean_	< median	44.0 months [33.1—NA]	0.07	53.3 months [36.6—NA]	0.023*	60.7 months [34.0—NA]	0.023*
	≥ median	17.5 months [12.5—NA]		15.4 months [11.9—NA]		14.5 months [10.2 – 52.0]	
SLR_max_	< median	38.4 months [27.4—NA]	0.69	48.9 months [31.3—NA]	0.18	35.3 months [18.2—NA]	0.71
	≥ median	22.8 months [13.3—NA]		16.2 months [11.9—NA]		29.6 months [11.0—NA]	
BLR_mean_	< median	36.6 months [21.2—NA]	0.84	62.7 months [38.3—NA]	0.013*	36.6 months [20.9—NA]	0.26
	≥ median	44.0 months [16.5—NA]		16.2 months [12.5 – 48.9]		25.0 months [13.5—NA]	
BLR_max_	< median	36.6 months [21.2—NA]	0.88	42.4 months [21.2—NA]	0.31	36.6 months [18.2—NA]	0.53
	≥ median	39.4 months [17.0—NA]		22.2 months [12.7—NA]		23.7 months [14.5—NA]	

*SLR*_*mean*_ spleen to liver SUV_mean_ ratio, *SLR*_*max*_ spleen to liver SUV_max_ ratio, *BLR*_*mean*_ bone marrow to liver SUV_mean_ ratio, *BLR*_*max*_ bone marrow to liver SUV_max_ ratio

^*^ Statistically significant difference

### Analysis of interim PET/CT findings

The findings of interim PET/CT were compared to those of the baseline scan and metabolic response evaluation was performed. The results of application of the metabolic criteria on interim PET/CT are presented in Table 4. With regard to survival analysis, no significant differences in OS were observed between the four metabolic response groups (CMR, PMR, SMD, PMD) according to EORTC (p = 0.31), PERCIST (p = 0.27), imPERCIST5 (p = 0.25) and iPERCIST (p = 0.27), although patients with CMR tended to have the longest OS. Based on the PERCIMT classification, a non-significant trend for longer OS was observed in patients with better metabolic response patterns to ICIs (p = 0.08) (Supplementary Fig. 1).

**Table 4:** Interim PET/CT response assessment to immunotherapy and survival analysis according to the applied metabolic criteria in 61 patients. Values refer to number of patients (%) and median OS [95% CI]

	EORTC		PERCIST		PERCIMT		imPERCIST5		iPERCIST	
	No. patients	OS (months)	No. patients	OS (months)	No. patients	OS (months)	No. patients	OS (months)	No. patients	OS (months)
PET/CT response										
CMR	3 (4.9%)	NA [NA—NA]	4 (6.6%)	NA [NA—NA]	3 (4.9%)	NA [NA—NA]	4 (6.6%)	NA [NA—NA]	4 (6.6%)	NA [NA—NA]
PMR	10 (16.4%)	NA [11.6—NA]	7 (11.5%)	62.7 [8.2—NA]	8 (13.1%)	NA [31.3—NA]	8 (13.1%)	62.7 [17.8—NA]	7 (11.5%)	62.7 [8.2—NA]
SMD	9 (14.8%)	20.0 [16.2—NA]	23 (37.7%)	35.3 [16.2—NA]	31 (50.8%)	22.2 [16.2—NA]	29 (47.5%)	31.3 [15.1—NA]	23 (37.7%)	35.3 [16.2—NA]
PMD	39 (63.9%)	19.9 [12.6—NA]	27 (44.3%)	15.4 [10.5—NA]	19 (31.1%)	12.6 [9.8—NA]	20 (32.8%)	15.4 [11.0—NA]	27 (44.3%)	15.4 [10.5—NA]
	*p* = *0.31*			*p* = *0.27*		*p* = *0.078*		*p* = *0.25*		*p* = *0.27*
Metabolic response rate (RR)										
Responders (CMR, PMR)	13 (21.3%)	NA [31.3—NA]	11 (18.0%)	62.7 [NA—NA]	11 (18.0%)	NA [NA—NA]	12 (20.2%)	62.7 [62.7—NA]	11 (18.0%)	62.7 [NA—NA]
Non-responders (SMD, PMD)	48 (78.7%)	19.9 [12.7 – 62.7]	50 (82.0%)	21.2 [12.7—NA]	50 (82.0%)	18.5 [12.6 – 62.7]	49 (80.0%)	21.2 [12.7—NA]	50 (82.0%)	21.2 [12.7—NA]
	*p* = *0.1*			*p* = *0.21*		*p* = 0.019*		*p* = *0.11*		*p* = *0.21*
Disease control rate										
Disease control (CMR, PMR, SMD)	22 (36.1%)	NA [17.8—NA]	34 (55.7%)	38.3 [22.2—NA]	42 (68.9%)	38.3 [21.2—NA]	41 (67.2%)	38.3 [21.2—NA]	34 (55.7%)	38.3 [22.2—NA]
No-disease control (PMD)	39 (63.9%)	19.9 [12.6 – NA]	27 (44.3%)	15.4 [10.5—NA]	19 (31.1%)	12.6 [9.8—NA]	20 (32.8%)	15.4 [11.0—NA]	27 (44.3%)	15.4 [10.5—NA]
	*p* = *0.17*			*p* = *0.23*		*p* = *0.084*		*p* = *0.28*		*p* = *0.23*

^*^ Statistically significant difference

The PMD results for iPERCIST refer to uPMD

**Fig. 2:**
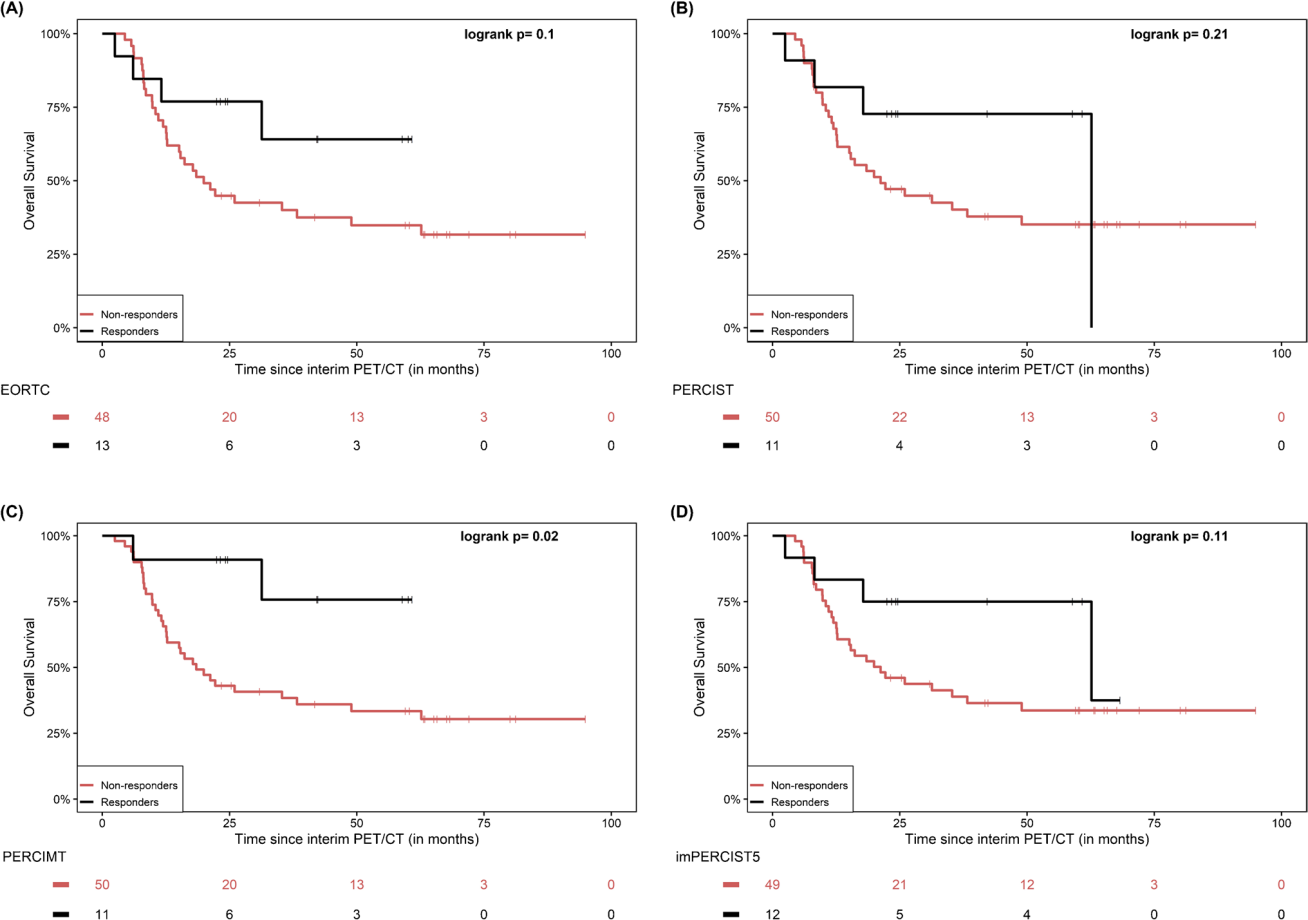
Kaplan–Meier estimates of OS based on the response rate “RR” dichotomization of patients in interim PET/CT according to the applied metabolic criteria EORTC (**A**), PERCIST (**B**), PERCIMT (**C**) and imPERCIST5 (**D**). The numbers of patients at risk in each group and for the respective time-points are shown below the plots. The Kaplan–Meier estimates based on iPERCIST are not presented since they are identical to PERCIST, with the exception that for description of metabolic progression the term uPMD is employed instead of PMD

When response to therapy was defined by metabolic response rate (“RR”), no significant differences between responders and non-responders were demonstrated according to EORTC (p = 0.1), PERCIST (p = 0.21), imPERCIST5 (p = 0.11) and iPERCIST (p = 0.21). On the other hand, the application of PERCIMT led to a significantly longer OS for patients classified as responders (p = 0.02) (Fig. 2).

**Fig. 3:**
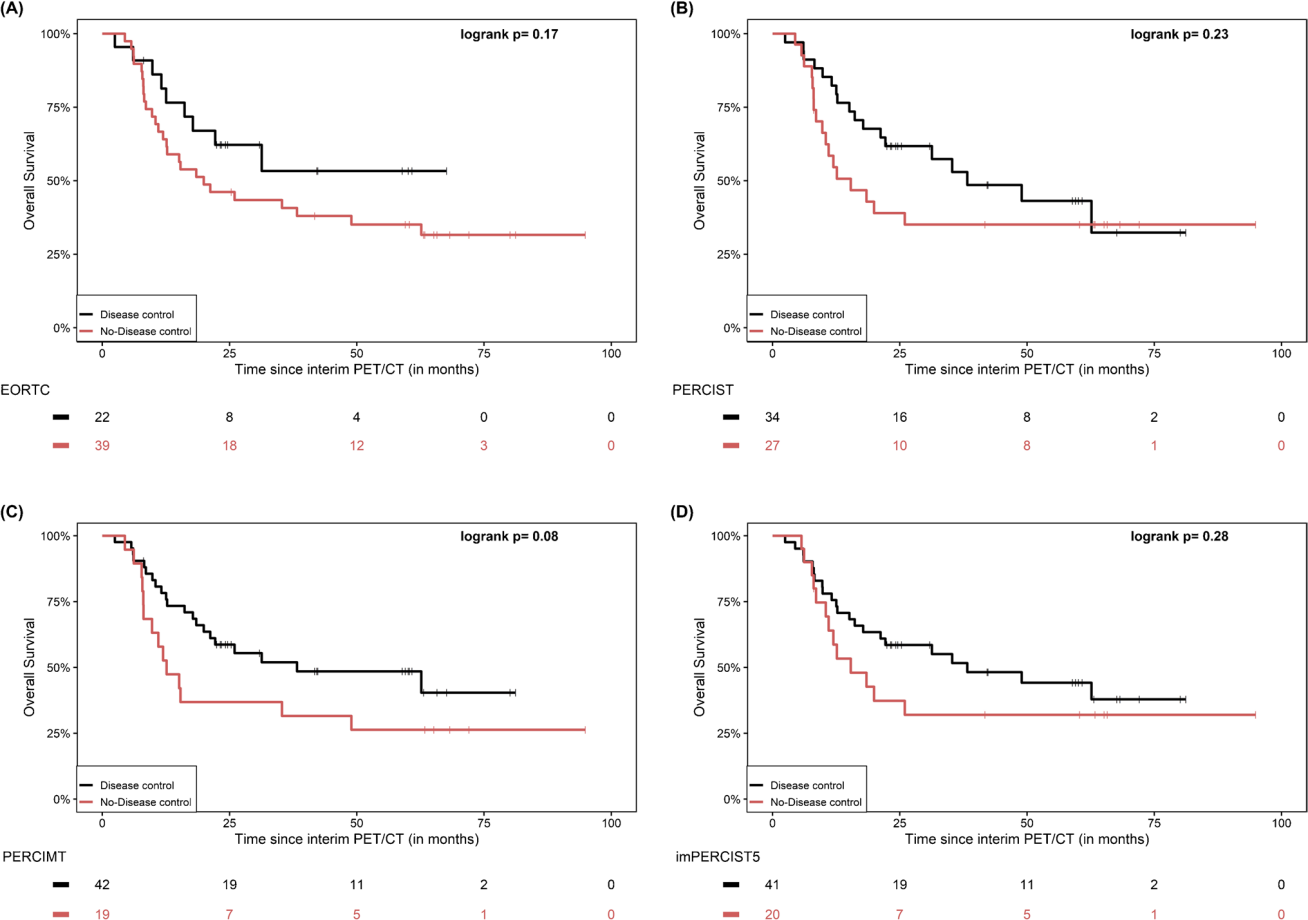
Kaplan–Meier estimates of OS based on the disease control rate “DCR” dichotomization of patients in interim PET/CT according to the applied metabolic criteria EORTC (**A**), PERCIST (**B**), PERCIMT (**C**) and imPERCIST5 (**D**). The numbers of patients at risk in each group and for the respective time-points are shown below the plots. The Kaplan–Meier estimates based on iPERCIST are not presented since they are identical to PERCIST, with the exception that for description of metabolic progression the term uPMD is employed instead of PMD

Based on the disease control rate (“DCR”) dichotomization, no significant differences between the disease control and no-disease control groups were observed according to EORTC (p = 0.17), PERCIST (p = 0.23), imPERCIST5 (p = 0.28) and iPERCIST (p = 0.23), while the application of PERCIMT revealed a non-significant trend for longer OS in patients with disease control (p = 0.08) (Fig. 3).

In total, 28 patients (41.8%) had PET/CT findings suggestive of irAEs on interim PET/CT, the most common of which being colitis. In terms of survival analysis, no significant difference in OS was observed between patients showing irAEs (median = 38.3 months, 95% CI = [11.9—NA]) and those without such signs (median = 31.3 months, 95% CI = [18.5—NA]) (p = 0.6).

The values of quantitative parameters derived from physiologic non-tumoral tissues on interim PET/CT are presented in Table 2. The respective survival analysis revealed a significantly longer OS for patients with lower than median SLR_mean_ (p = 0.02) and BLR_mean_ (p = 0.01) (Table 3) (Fig. 1B).

### Analysis of late PET/CT findings

The findings of late PET/CT were also compared to the baseline scan after application of the respective metabolic criteria (Table 5). Survival analysis revealed significantly longer OS for patients with better metabolic response pattern based on PERCIMT (p = 0.046), and a respective non-significant trend also for PERCIST (p = 0.07) and iPERCIST (p = 0.07). However, such differences were not observed after application of EORTC (p = 0.19) and imPERCIST5 (p = 0.11) (Supplementary Fig. 2).

**Table 5:** Late PET/CT response assessment to immunotherapy and survival analysis according to the applied metabolic criteria in 61 patients. Values refer to number of patients (%) and median OS [95% CI]

	EORTC		PERCIST		PERCIMT		imPERCIST5		iPERCIST	
	No. patients	OS (months)	No. patients	OS (months)	No. patients	OS (months)	No. patients	OS (months)	No. patients	OS (months)
PET/CT response										
CMR	8 (13.1%)	NA [23.7—NA]	9 (14.8%)	NA [NA—NA]	8 (13.1%)	NA [23.7—NA]	8 (13.1%)	NA [NA—NA]	9 (14.8%)	NA [NA—NA]
PMR	12 (19.7%)	60.7 [10.2—NA]	5 (8.2%)	60.7 [15.6—NA]	13 (21.3%)	60.7 [10.2—NA]	7 (11.5%)	60.7 [10.2—NA]	5 (8.2%)	60.7 [15.6—NA]
SMD	6 (9.8%)	20.3 [15.6—NA]	13 (21.3%)	NA [20.3—NA]	19 (31.1%)	20.3 [14.5—NA]	23 (37.7%)	14.5 [9.3—NA]	13 (21.3%)	NA [20.3—NA]
PMD	35 (57.4%)	13.7 [9.8—NA]	34 (55.7%)	13.5 [9.3 – 47.4]	21 (34.4%)	10.6 [6.5 – NA]	23 (37.7%)	18.2 [11.0—NA]	34 (55.7%)	13.5 [9.3 – 47.4]
	*p* = *0.19*			*p* = *0.066*		*p* = 0.046*		*p* = *0.11*		*p* = *0.066*
Metabolic response rate (RR)										
Responders (CMR, PMR)	20 (32.8%)	60.7 [23.7—NA]	14 (23.0%)	60.7 [60.7—NA]	21 (34.4%)	60.7 [29.6—NA]	15 (24.6%)	60.7 [60.7—NA]	14 (23.0%)	60.7 [60.7—NA]
Non-responders (SMD, PMD)	41 (67.2%)	15.6 [10.9 – NA]	47 (77.0%)	18.2 [11.0 – NA]	40 (65.6%)	14.5 [10.9 – 47.4]	46 (75.4%)	16.8 [11.0 – 47.4]	47 (77.0%)	18.2 [11.0 – NA]
	*p* = *0.08*			*p* = *0.082*		*p* = 0.042*		*p* = 0.043*		*p* = *0.082*
Disease control rate										
Disease control (CMR, PMR, SMD)	26 (42.6%)	60.7 [23.7—NA]	27 (44.3%)	60.7 [23.7—NA]	40 (65.6%)	60.7 [20.9—NA]	38 (62.3%)	29.6 [14.5—NA]	27 (44.3%)	60.7 [23.7—NA]
No-disease control (PMD)	35 (57.4%)	13.7 [9.8—NA]	34 (55.7%)	13.5 [9.3 – 47.4]	21 (34.4%)	10.6 [6.5 – NA]	23 (37.7%)	18.2 [11.0—NA]	34 (55.7%)	13.5 [9.3 – 47.4]
	*p* = *0.053*			*p* = 0.012*		*p* = 0.01*		*p* = *0.67*		*p* = 0.012*

^*^ Statistically significant difference

The PMD results for iPERCIST refer to cPMD

**Fig. 4:**
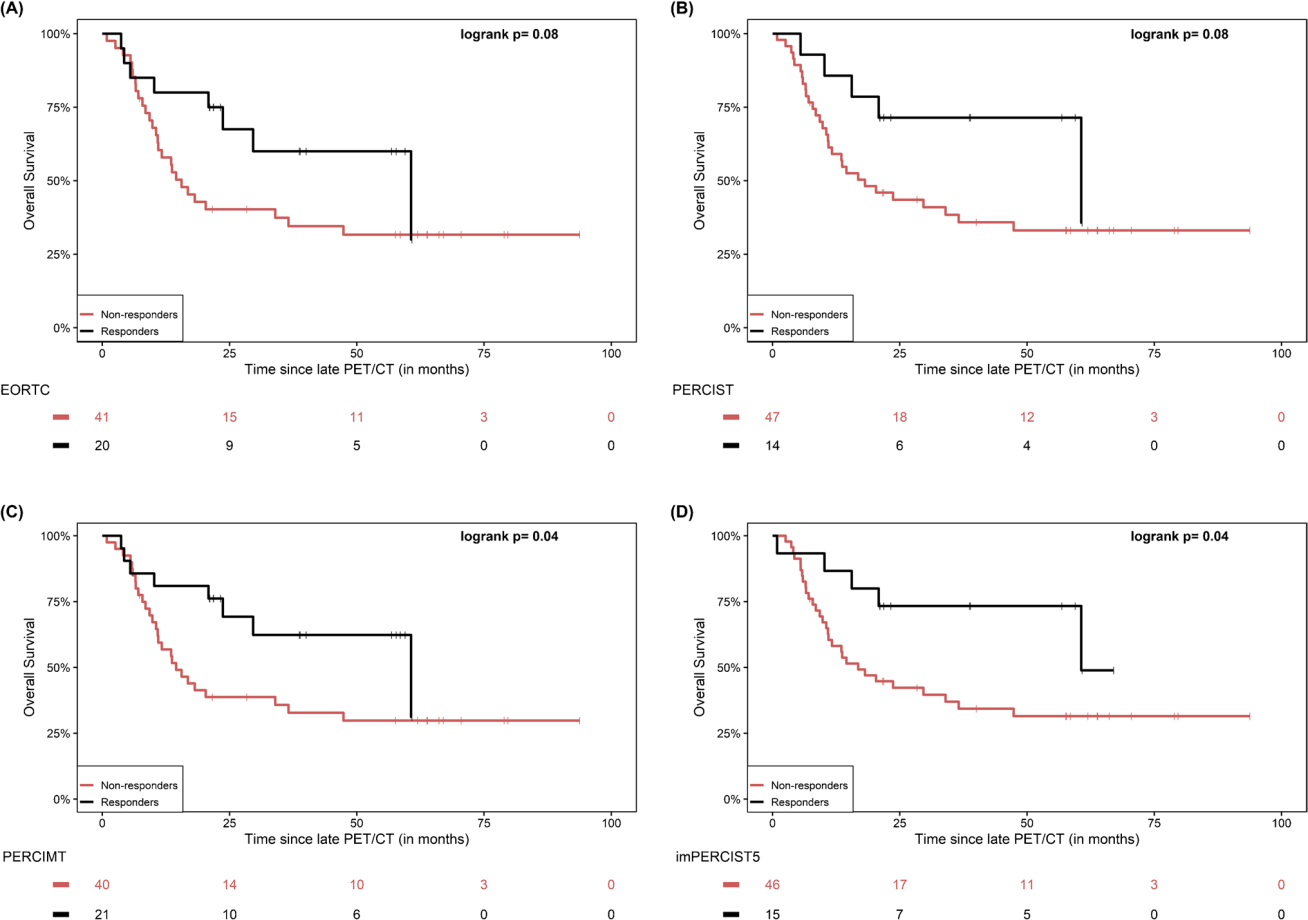
Kaplan–Meier estimates of OS based on the response rate “RR” dichotomization of patients in late PET/CT according to the applied metabolic criteria EORTC (**A**), PERCIST (**B**), PERCIMT (**C**) and imPERCIST5 (**D**). The numbers of patients at risk in each group and for the respective time-points are shown below the plots. The Kaplan–Meier estimates based on iPERCIST are not presented since they are identical to PERCIST, with the exception that for description of metabolic progression the term cPMD is employed instead of PMD

When response to therapy was defined by metabolic response rate (“RR”), a significantly longer OS was observed in responders compared to non-responders according to PERCIMT (p = 0.04) and imPERCIST5 (p = 0.04), while a similar non-significant trend for longer OS was also observed according to EORTC (p = 0.08), PERCIST (p = 0.08) and iPERCIST (p = 0.08) (Fig. 4).

**Fig. 5:**
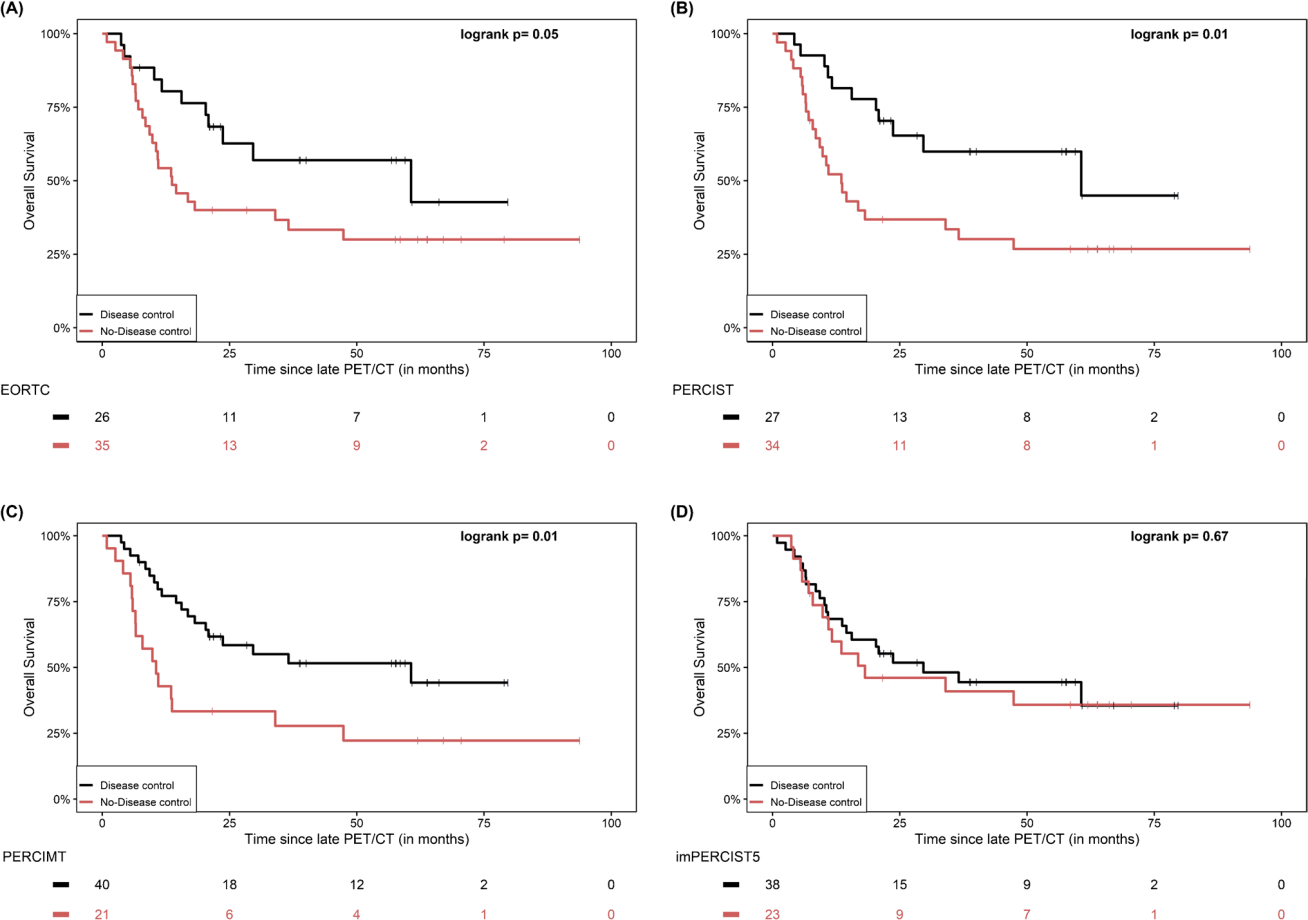
Kaplan–Meier estimates of OS based on the disease control rate “DCR” dichotomization of patients in late PET/CT according to the applied metabolic criteria EORTC (**A**), PERCIST (**B**), PERCIMT (**C**) and imPERCIST5 (**D**). The numbers of patients at risk in each group and for the respective time-points are shown below the plots. The Kaplan–Meier estimates based on iPERCIST are not presented since they are identical to PERCIST, with the exception that for description of metabolic progression the term cPMD is employed instead of PMD

Further, when patients were dichotomized based on the disease control rate (“DCR”), a significantly longer OS was exhibited in the disease control compared to no-disease control group according to PERCIST (p = 0.01), PERCIMT (p = 0.01) and iPERCIST (p = 0.01), while a similar, marginally non-significant trend was also observed after application of EORTC (p = 0.05). In contrary, the application of imPERCIST5 led to no significant differences between the two groups (p = 0.67) (Fig. 5).

Thirty-six patients (53.7%) had PET/CT findings suggestive of irAEs on late PET/CT, with colitis being the most common one. The aggregated results regarding frequency of occurrence of PET signs of irAEs during immunotherapy are provided in Table 6. Similar to interim PET/CT, the dichotomisation of the patient cohort based on the emergence of irAEs (median = 47.4 months, 95% CI = [23.7—NA]) or not (median = 18.2 months, 95% CI = [11.7—NA]) revealed no significant difference in OS (p = 0.2). The Kaplan–Meier estimates of OS according to the emergence of radiologic irAEs on interim and late PET/CT are presented in Supplementary Fig. 3.

**Table 6:** Frequency of occurrence of irAEs during immunotherapy on interim and late PET/CT. In the cells the number of patients developing each adverse event is indicated

	Interim PET/CT	Late PET/CT
Colitis	19	24
Gastritis	1	1
Duodenitis	1	-
Bone marrow activation	8	11
Arthritis	3	6
Thyroiditis	3	2
Pneumonitis	1	1
Sarcoid-like lymphadenopathy	3	3

The values of the quantitative parameters derived from physiologic non-tumoral tissues on late PET/CT imaging are presented in Table 2. Survival analysis revealed a significantly longer OS for patients with lower SLR_mean_ values (*p* = 0.02) (Fig. 1C). The aggregated results of survival analysis based on quantitative data from physiologic non-tumoral tissues are presented in Table 3.

## Discussion

In this study we investigated the prognostic value of [^18^F]FDG PET/CT as part of response monitoring in metastatic melanoma patients treated with ICIs. The main findings of our analysis are the following: firstly, metabolic response based on [^18^F]FDG PET/CT, performed after four cycles of immunotherapy, is significantly associated with OS after application of different metabolic criteria. Secondly, the modality also carries significant prognostic information on patient survival already after administration of the first two ICIs’ cycles, especially with employment of the novel immunotherapy-modified PERCIMT. Thirdly, the assessment of immune activation signs in the spleen may have a potential role as prognosticator, as reflected by the longer survival of patients exhibiting a low SLR_mean_ throughout the course of immunotherapy. On the other hand, the emergence of PET signs of irAEs is not associated with patient survival.

The identification of appropriate time points and methodologies for the reliable assessment of response to immunotherapy is one of the main challenges in molecular imaging. According to the recently published joint EANM/SNMMI/ANZSNM guidelines [24], [^18^F]FDG PET/CT should be considered mandatory at baseline for tumor assessment before start of immunotherapy. It is, moreover, recommended at approximately 8–12 weeks (i.e. 3–4 cycles) after treatment start as well as in patients requiring a temporary interruption of immunotherapy. Regarding the most appropriate approach for the interpretation of response to ICIs, although there is yet insufficient evidence to decide whether the predictive value of the immunotherapy-modified PET/CT criteria is superior to conventional ones [29], as general recommendation, in case doubts exist between progression or pseudoprogression, a confirmatory follow-up [^18^F]FDG PET/CT study 4–8 weeks later should be performed [24]. This approach is in line with the general principle of iRECIST [30] and iPERCIST [18], which by definition require a confirmation of an initial progressive disease with follow-up imaging.

We, herein, performed [^18^F]FDG PET/CT at baseline and at two different time points during follow-up, namely after administration of two and four cycles of ICIs. The main rationale behind applying PET imaging already after the first two immunotherapy cycles is the significance of early detection of non-responders that are unlikely to profit from ICIs. These patients would potentially benefit from an early cessation of a non-effective treatment and a change in therapeutic management at the appropriate time, while limiting the treatment-associated toxicity and financial burden [31]. At this early time point, response on PET/CT as assessed by the conventional metabolic criteria (EORTC, PERCIST) did not significantly correlate with patient survival. On the other hand, the application of the novel, immunotherapy-modified PERCIMT revealed significantly longer OS for responders (CMR, PMR) compared to non-responders (SMD, PMD) (*p* < 0.05) as well as a non-significant trend for longer OS in patients with metabolic disease control (CMR, PMR, SMD) compared to PMD (*p* < 0.1). These results are in agreement with previous findings by our group that highlighted the higher performance of PERCIMT over EORTC, which mainly stems from the different approach for definition of PMD between the criteria [32, 33]. In view of these, although the need for early, reliable assessment of response to immunotherapy is evident, our results call for careful handling of early PET/CT findings, especially those indicating metabolic progression, as the early diagnosis of progressive disease carries the risk of misdiagnosis—due to the phenomenon of pseudoprogression—of some late responders as PMD, thus depriving these patients of the beneficial effect of immunotherapy. In this context, the performance of another follow-up PET/CT at a second time-point for confirmation of early PMD, especially in the most ambiguous cases, as recommended by the EANM/SNMMI/ANZSNM guidelines, represents a reasonable approach.

**Fig. 6:**
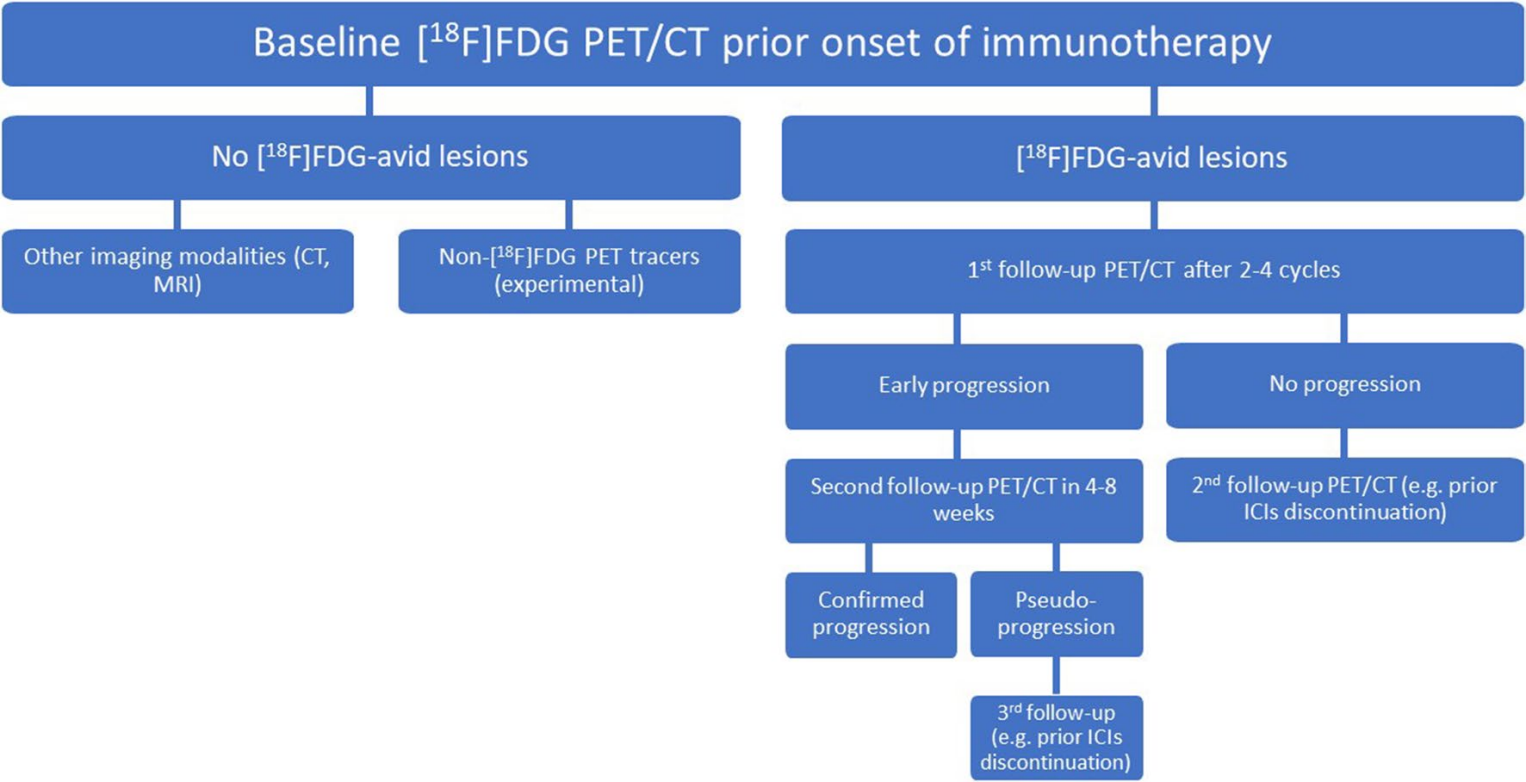
Flowchart of a proposed approach for employment of [^18^F]FDG PET/CT at different time points during immunotherapy

The predictive value of [^18^F]FDG PET/CT for OS was higher when applied later during treatment, in specific after administration of four ICIs cycles. At that time point, metabolic responders according to the RR dichotomization showed a trend for longer survival according to the conventional PET criteria (EORTC, PERCIST; *p* < 0.1), which, moreover, was statistically significant with the immunotherapy-modified PERCIMT and imPERCIST5 (*p* < 0.05). Respectively, when response to immunotherapy was defined by metabolic DCR, patients exhibiting disease control had a significantly longer survival than those with disease progression according to almost all metabolic criteria. These results come to substantiate previous works in the field reflecting the ability of metabolic response to ICIs, as assessed by [^18^F]FDG PET/CT performed approximately 3–4 months after starting treatment, in stratifying longer-term patient outcome [23, 34]. This time point for performing follow-up imaging appears sufficiently reliable, irrespective of the evaluation method used, and practical in terms of patient management. On the basis of these findings, serial [^18^F]FDG PET/CT scanning during immunotherapy seems to be justified, especially in view of potential clinical decisions on the continuation or not of immunotherapy (Fig. 6) [35].

An interesting finding in our analysis was the lack of identifiable target lesion(s) on baseline PET/CT according to the PERCIST and PERCIST-based (imPERCIST5, iPERCIST) approaches, despite the identification of such lesions with visual reading of the scans, in 16 patients. Nevertheless, these patients were included in the metabolic response evaluations, as suggested by the authors of PERCIST, since disease progression can be designated even if the target lesion at baseline does not meet the minimum threshold for evaluation [36]. Notably, the exclusion of these 16 patients from the serial metabolic response analysis would have a positive impact on the performance of these criteria both in interim and late PET/CT, increasing the survival differences between the various patient response groups (partly at a statistically significant level), which reflects the higher performance of PERCIST when there are already identified target lesions at baseline (Supplementary Fig. 4–9). The observed differentiation between the applied criteria regarding the identification of target lesion(s) is consistent with a prior study assessing treatment response to lenvatinib in thyroid cancer by means of [^18^F]FDG PET/CT. This study reported a proportion of 20% of patients with no identifiable target lesions according to the PERCIST-based approaches in comparison to EORTC [37]. The reason for this finding is apparently related to the differences in definition of target lesion(s) by the various metabolic criteria: PERCIST precisely define target lesions, requiring these to demonstrate a SUL_peak_ at least 1.5 times greater than the minimum background, which is set at the SUL_mean_ + 2SDs of the normal liver parenchyma. In contrary, the EORTC (and the PERCIMT) criteria do not define a minimum background, rendering smaller and less metabolically active tumor lesions (depending on the surrounding tissue) eligible for evaluation, thus, allowing their inclusion in response assessment. In this framework, EORTC, and subsequently PERCIMT, may prove easier to apply in everyday clinical practice regarding response assessment to immunotherapy. On the other hand, the more ‘vague’ definition of target lesions proposed by EORTC risks being subject to non-negligible inter-observer variability, leading to reasonable concerns about its reliability.

Apart from the assessment of treatment response based on the evaluation of tumor lesions, our analysis also involved the investigation of signs of immune activation on PET as a supplementary approach for prediction of the immunotherapy effect. We, specifically, performed semi-quantitative calculations of [^18^F]FDG uptake in two immune organs, namely the spleen and the bone marrow, and derived spleen-to-liver (SLR) and bone marrow-to-liver (BLR) ratios. Herein, the principal finding was the association between SLR_mean_ and patient survival. In particular, patients with lower SLR_mean_ values exhibited a longer OS, a result that was consistent throughout the duration of follow-up. Ascertaining the role of spleen metabolism during immunotherapy has proved to be a challenging task, with the so far published results on the interpretation of this phenomenon being rather inconclusive. Indeed, on the one hand, the present results are in line with previous works [22, 33, 38] suggesting that an increased spleen metabolism on [^18^F]FDG PET/CT correlates with an unfavorable patient outcome. On the other hand, in a prospective study of [^18^F]FDG PET/MRI in melanoma patients under ICIs, a significant increase in spleen metabolism (SUL_mean_) was observed as early as two weeks after treatment initiation in responders, in contrary to non-responders who showed stability of the parameter [39]. In this context, although our results highlight the potential role of SLR_mean_ as prognosticator, they should be interpreted with caution. Overall, however, the investigation by functional imaging not only of the tumor but also the host’s immune system is gradually gaining importance as a potential surrogate marker of therapeutic response. Future prospective studies should determine whether this approach could indeed serve as a prognostic indicator of immunotherapy.

Driven by the reported association between the emergence of irAEs and the anti-tumor effect elicited by ICIs [10-12], we also investigated the emergence of PET signs indicative of irAEs during the course of immunotherapy and their potential correlation with patient outcome. Given the lack of a standard definition for immunotherapy-induced organ inflammation in PET/CT, these signs were documented based on visual, thus per se subjective, assessments of patient scans. Interestingly, a large proportion of patients demonstrated irAEs on interim (42%) and late (54%) imaging, the most common being colitis, which is a documented PET finding in melanoma patients mainly under ipilimumab [40]. However, the presence of these signs did not have a significant effect on OS, which is in line with the available, rather non-conclusive evidence in the field [14].

Our study has some limitations. Firstly, this is a single center, retrospective analysis of prospectively acquired data. This resulted in the lack of certain evidence, such as clinical and laboratory data for further investigation of PET signs of irAEs or other signs suggestive of immune activation during treatment. Thus, a validation of the herein presented findings in larger patient cohorts ideally studied in the context of a multicenter, prospective trial would be required. Another limitation lies in the fact that most patients (73%) in the present series were treated with ipilimumab monotherapy, which is no longer the standard of care in melanoma; instead, first-line melanoma treatment nowadays involves anti-PD-1 monoclonal antibodies, applied both as single agents and in combination with ipilimumab. These treatments were administered to the rest 27% of the cohort. However, it is noteworthy that the majority of the novel immunotherapy-modified criteria have been derived from patient cohorts treated with ipilimumab. Further, the vast majority of positive PET/CT findings were not histopathologically confirmed, which, however, is not possible in the clinical setting.

## Conclusions

This study provides data on the relationship of metabolic features on [^18^F]FDG PET/CT applied at different, strictly defined time points during immunotherapy and survival of patients with metastatic melanoma treated with ICIs. Our results demonstrate a significant association between response on [^18^F]FDG PET/CT, as assessed by different metabolic criteria, and OS, especially when the modality is performed after four cycles of immunotherapy. Moreover, [^18^F]FDG PET/CT carries significant prognostic information on patient survival already after the administration of first two ICIs’ cycles, a time-point in which the employment of novel immunotherapy-modified criteria should be considered. Finally, our data provide further evidence on the potential role of spleen metabolism as a prognosticator in melanoma patients under immunotherapy.

## Supplementary Information

Below is the link to the electronic supplementary material.

Supplementary file1 (DOCX 8147 KB)

## Data Availability

The datasets generated during and/or analysed during the current study are available from the corresponding author on reasonable request.
