# Supplementary material for: The prognostic value of [18F]FDG PET/CT based response monitoring in metastatic melanoma patients undergoing immunotherapy: comparison of different metabolic criteria
Source: Eur J Nucl Med Mol Imaging. 2023 Apr 26;50(9):2699–714. doi: 10.1007/s00259-023-06243-y (PMC10317882; doi:10.1007/s00259-023-06243-y)
Supplement: Supplementary file 1 — Supplementary file1 (DOCX 8147 KB) [file 259_2023_6243_MOESM1_ESM.docx]

**Supplementary Figure 1** Kaplan-Meier estimates of OS based on the different metabolic response patterns (CMR, PMR, SMD, PMD) identified in interim PET/CT according to the applied PET/CT criteria EORTC (A), PERCIST (B), PERCIMT (C) and imPERCIST5 (D). The numbers of patients at risk in each group and for the respective time-points are shown below the plots. The Kaplan-Meier estimates based on iPERCIST are not presented since they are identical to PERCIST, with the exception that for description of metabolic progression the term uPMD is employed instead of PMD.





**Supplementary Figure 2** Kaplan-Meier estimates of OS based on the different metabolic response patterns (CMR, PMR, SMD, PMD) identified in late PET/CT according to the applied PET/CT criteria EORTC (A), PERCIST (B), PERCIMT (C) and imPERCIST5 (D). The numbers of patients at risk in each group and for the respective time-points are shown below the plots. The Kaplan-Meier estimates based on iPERCIST are not presented since they are identical to PERCIST, with the exception that for description of metabolic progression the term cPMD is employed instead of PMD.

**

**

**Supplementary Figure 3** Kaplan-Meier estimates of OS according to the emergence of radiologic irAEs on interim (A) and late (B) PET/CT. The numbers of patients at risk in each group and for the respective time-points are shown below the plots.


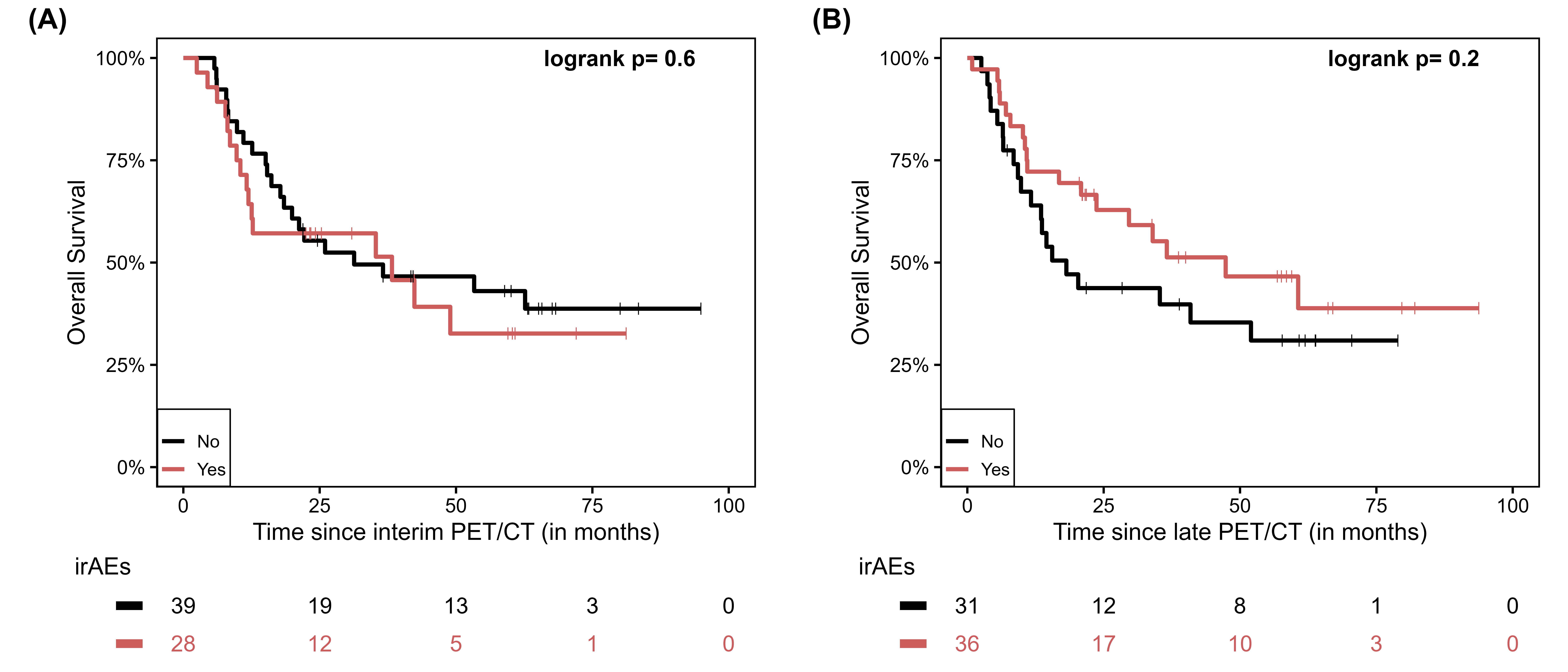


**Supplementary Figure 4** Kaplan-Meier estimates of OS based on the different metabolic response patterns (CMR, PMR, SMD, PMD) identified in interim PET/CT according to the applied PET/CT criteria EORTC (A), PERCIST (B), PERCIMT (C) and imPERCIST5 (D). The assessments of PERCIST and imPERCIST5 have been performed after exclusion of the 16 patients without identifiable target lesion(s) on baseline PET/CT according to the target lesion definition of these criteria. The numbers of patients at risk in each group and for the respective time-points are shown below the plots. The Kaplan-Meier estimates based on iPERCIST are not presented since they are identical to PERCIST, with the exception that for description of metabolic progression the term uPMD is employed instead of PMD.





**Supplementary Figure 5** Kaplan-Meier estimates of OS based on the response rate “RR” dichotomization of patients in interim PET/CT according to the applied metabolic criteria EORTC (A), PERCIST (B), PERCIMT (C) and imPERCIST5 (D). The assessments of PERCIST and imPERCIST5 have been performed after exclusion of the 16 patients without identifiable target lesion(s) on baseline PET/CT according to the target lesion definition of these criteria. The numbers of patients at risk in each group and for the respective time-points are shown below the plots. The Kaplan-Meier estimates based on iPERCIST are not presented since they are identical to PERCIST, with the exception that for description of metabolic progression the term uPMD is employed instead of PMD.





**Supplementary Figure 6** Kaplan-Meier estimates of OS based on the disease control rate “DCR” dichotomization of patients in interim PET/CT according to the applied metabolic criteria EORTC (A), PERCIST (B), PERCIMT (C) and imPERCIST5 (D). The assessments of PERCIST and imPERCIST5 have been performed after exclusion of the 16 patients without identifiable target lesion(s) on baseline PET/CT according to the target lesion definition of these criteria. The numbers of patients at risk in each group and for the respective time-points are shown below the plots. The Kaplan-Meier estimates based on iPERCIST are not presented since they are identical to PERCIST, with the exception that for description of metabolic progression the term uPMD is employed instead of PMD.





**Supplementary Figure 7** Kaplan-Meier estimates of OS based on the different metabolic response patterns (CMR, PMR, SMD, PMD) identified in late PET/CT according to the applied PET/CT criteria EORTC (A), PERCIST (B), PERCIMT (C) and imPERCIST5 (D). The assessments of PERCIST and imPERCIST5 have been performed after exclusion of the 16 patients without identifiable target lesion(s) on baseline PET/CT according to the target lesion definition of these criteria. The numbers of patients at risk in each group and for the respective time-points are shown below the plots. The Kaplan-Meier estimates based on iPERCIST are not presented since they are identical to PERCIST, with the exception that for description of metabolic progression the term uPMD is employed instead of PMD.





**Supplementary Figure 8** Kaplan-Meier estimates of OS based on the response rate “RR” dichotomization of patients in late PET/CT according to the applied metabolic criteria EORTC (A), PERCIST (B), PERCIMT (C) and imPERCIST5 (D). The assessments of PERCIST and imPERCIST5 have been performed after exclusion of the 16 patients without identifiable target lesion(s) on baseline PET/CT according to the target lesion definition of these criteria. The numbers of patients at risk in each group and for the respective time-points are shown below the plots. The Kaplan-Meier estimates based on iPERCIST are not presented since they are identical to PERCIST, with the exception that for description of metabolic progression the term uPMD is employed instead of PMD.





**Supplementary Figure 9** Kaplan-Meier estimates of OS based on the response rate “DCR” dichotomization of patients in late PET/CT according to the applied metabolic criteria EORTC (A), PERCIST (B), PERCIMT (C) and imPERCIST5 (D). The assessments of PERCIST and imPERCIST5 have been performed after exclusion of the 16 patients without identifiable target lesion(s) on baseline PET/CT according to the target lesion definition of these criteria. The numbers of patients at risk in each group and for the respective time-points are shown below the plots. The Kaplan-Meier estimates based on iPERCIST are not presented since they are identical to PERCIST, with the exception that for description of metabolic progression the term uPMD is employed instead of PMD.
